# The need for clinical studies assessing whether weight loss improves functional outcome after stroke in diabetes

**DOI:** 10.1186/s12916-026-04809-9

**Published:** 2026-03-24

**Authors:** Ellen Vercalsteren, Michael V. Mazya, Thomas Nyström, Vladimer Darsalia, Cesare Patrone

**Affiliations:** 1 NeuroCardioMetabol Group, Department of Clinical Science and Education, Södersjukhuset, Internal Medicine, Karolinska Institutet, Stockholm, 118 83 Sweden; 2 Department of Neurology, Department of Clinical Neuroscience, Karolinska University Hospital, Karolinska Institutet, Stockholm, 171 76 Sweden

**Keywords:** Stroke, Diabetes, Obesity, Weight loss, Glucagon-like peptide 1 receptor agonists

## Abstract

**Background:**

Type 2 diabetes worsens functional outcome after stroke, severely affecting the rehabilitation processes, with no therapy available for this medical problem.

Main text.

Weight loss is an effective strategy for managing type 2 diabetes, with some studies also showing that it can reduce cardiovascular and stroke risk in this population. Recent animal studies suggest that weight loss induced by a diet change or pharmacologically (via the activation of the glucagon-like peptide 1 receptor) also improves functional outcome after stroke. Today, however, no clinical study has yet addressed this question.

This issue is important to address since type 2 diabetes is one of the strongest risk factors for stroke and the growing prevalence of diabetes is leading to an increasing number of stroke patients with type 2 diabetes who will require effective therapies. Here, we discuss recent findings showing the positive effects of weight loss in type 2 diabetes and its cardiovascular complications, underlining the need to perform new clinical studies specifically focused on understanding the potential therapeutic role of weight loss to improve functional outcomes after stroke.

**Conclusions:**

In summary, this debate underscores a critical clinical gap in current post-stroke care strategies and highlights the potential for weight loss as a novel treatment paradigm to improve functional stroke outcomes in type 2 diabetes. If validated in clinical studies, this approach will significantly improve the quality of life of many stroke patients with type 2 diabetes.

## Background

### Type 2 diabetes and stroke

Type 2 diabetes (T2D) is a metabolic disease characterized by hyperglycemia, overweight, and insulin resistance. T2D worsens neurological outcome after stroke and is a strong predictor of persistent post-stroke functional impairment [1–4], significantly increasing the stroke-related disability burden [5, 6]. Since T2D is also a strong risk factor for stroke [7–9] and T2D prevalence is estimated to reach 700 million by 2045 [10], this creates a large population in need of therapies to prevent stroke and reduce stroke-related disability.

## Main text

### Obesity and stroke

Obesity, clinically defined as a body mass index (BMI) of 30 kg/m^2^ or higher, affects more than 890 million (13%) adults globally (World Health Organization; WHO; (https://www.who.int/en/news-room/fact-sheets/detail/obesity-and-overweight).

Obesity is a major independent risk factor for developing T2D, with about 90% of people with T2D being obese or overweight (BMI of at least 25; http://www.who.int/dietphysicalactivity/media/en/gsfs_obesity.pdf). Moreover, obesity contributes directly to cardiovascular risk factors such as dyslipidemia, T2D, and hypertension leading to cardiovascular disease (CVD) and CVD-related mortality independently of other cardiovascular risk factors [11]. When considering stroke risk specifically, obesity is one of the most important modifiable risk factors for ischemic stroke [12], despite a so-called obesity paradox in which BMI has an inverse association with stroke risk in T2D [13]. Moreover, obesity has been linked to better functional outcomes in patients suffering a stroke [14]. This could possibly be secondary to acute anti-inflammatory effects immediately after stroke [15], specific stroke subtypes [16], and a short-term metabolic reserve [17]. However, recently, the accuracy of BMI to define and diagnose obesity has been put into question, as it fails to completely capture the complexity of the disease [18, 19]. Interestingly, studies investigating the effect of obesity on stroke outcomes using more precise definitions for obesity have demonstrated that increased visceral adiposity worsens stroke outcomes [20, 21]. Moreover, BMI has been shown to have a U- or J-shaped association to stroke-related disability and stroke-related quality of life [22], resulting in a strong contribution of high BMI to stroke-related disability globally [14, 23].

### Weight loss for the prevention and remission of type 2 diabetes

Given the close relationship between obesity and T2D, it is not surprising that weight loss strategies have been developed to both reduce the risk of developing T2D and to treat T2D in people with obesity.

Weight loss through lifestyle changes can reduce the incidence of T2D in individuals with obesity at high risk of T2D [24]. Moreover, professionally supported intensive weight management in the DiRECT study led to nearly half of participants with T2D achieving remission, defined as a return to non-diabetic status without the use of antidiabetic drugs after 12 months [25]. Finally, although safety remains an important consideration, strong evidence indicates that bariatric procedures result in even greater improvements than nonsurgical interventions in both the prevention and treatment of T2D [26–28]. Of note, the potential for sarcopenia and malnutrition associated with any weight loss strategy [29, 30] underscores the importance of multicomponent weight loss strategies incorporating nutritional and exercise interventions to mitigate the risk for these adverse outcomes [31, 32].

### Lack of clinical evidence linking weight loss to stroke severity and recovery in T2D

#### Lifestyle strategies and bariatric surgery

Weight loss strategies based on lifestyle changes showed moderate effects on the incidence of cardiovascular disease in participants with T2D in the Look AHEAD study [33]. Meanwhile, in the same study, patients with T2D remission experienced a 40% reduction in CVD risk [34]. Plausibly, as indicated by secondary analyses of the ‘Look AHEAD’ study and of the DiRECT and DIADEM-1 trial, weight loss must reach a certain magnitude to decrease CVD risk [33, 35].

Moreover, several recent studies demonstrate long-term reductions in CVD risk with sustained weight loss [36, 37]. Growing evidence indicates that bariatric surgery also reduces the risk of both microvascular and macrovascular complications of T2D compared with standard medical care, as reviewed by Arterburn and colleagues [27]. Most of these studies focused on overall CVD risk and did not specifically address stroke risk as the outcome, and those focusing on stroke risk specifically provided contradictory results. However, a recent review analyzing 24 studies involving 5,798,826 subjects concluded that lowering BMI reduces stroke risk in overweight/obese individuals [38]. Remarkably, studies on the effects of lifestyle/weight loss or of bariatric surgery on functional outcomes after stroke (in participants with or without T2D) cannot be found in literature (Search terms used in Pubmed: “stroke AND outcome AND weight loss” + “stroke AND neurological recovery AND weight loss”, “stroke AND outcome AND obesity” + “stroke AND neurological recovery AND obesity”, “stroke AND outcome AND diabetes” + “stroke AND neurological recovery AND diabetes”; no limit in time).

#### Glucagon-like peptide 1 receptor agonists (GLP-1RA)

In recent years T2D management has been revolutionized by novel treatments targeting the GLP-1R. GLP-1RA exert potent effects on glucose metabolism through glucose-dependent insulin secretion and promote weight loss via anorexic mechanisms [39]. Given the demonstrated efficacy of these treatments on T2D and weight, several studies have investigated their potential efficacy to prevent CVD. Indeed, GLP-1RA can reduce CVD risk in obese individuals both with and without T2D (SUSTAIN-6 study [40], SELECT trial [41]). Interestingly, in SUSTAIN-6 the 26% lower risk of the primary CVD composite outcome induced by the GLP-1RA semaglutide was principally driven by a significant (39%) decrease in nonfatal stroke [40], albeit that stroke risk specifically was not investigated. Moreover, it remains unclear whether weight loss was the main or only driving factor behind the efficacy of these drugs in reducing CVD risk. Indeed, recent studies demonstrate that the reduced CVD risk with GLP-1RA was not attributable only to weight loss [42, 43]. Moreover, GLP-1RA exert direct acute neuroprotective effects, further underpinning their potential to improve stroke neurological outcome by reducing stroke severity [44, 45].

Remarkably, none of the GLP-1RA studies have addressed stroke risk specifically, and no clinical studies investigating the effects of these drugs on outcomes after stroke can be found in the literature.

### The need for clinical studies investigating whether targeting obesity in T2D improves stroke outcomes

**Fig. 1:**
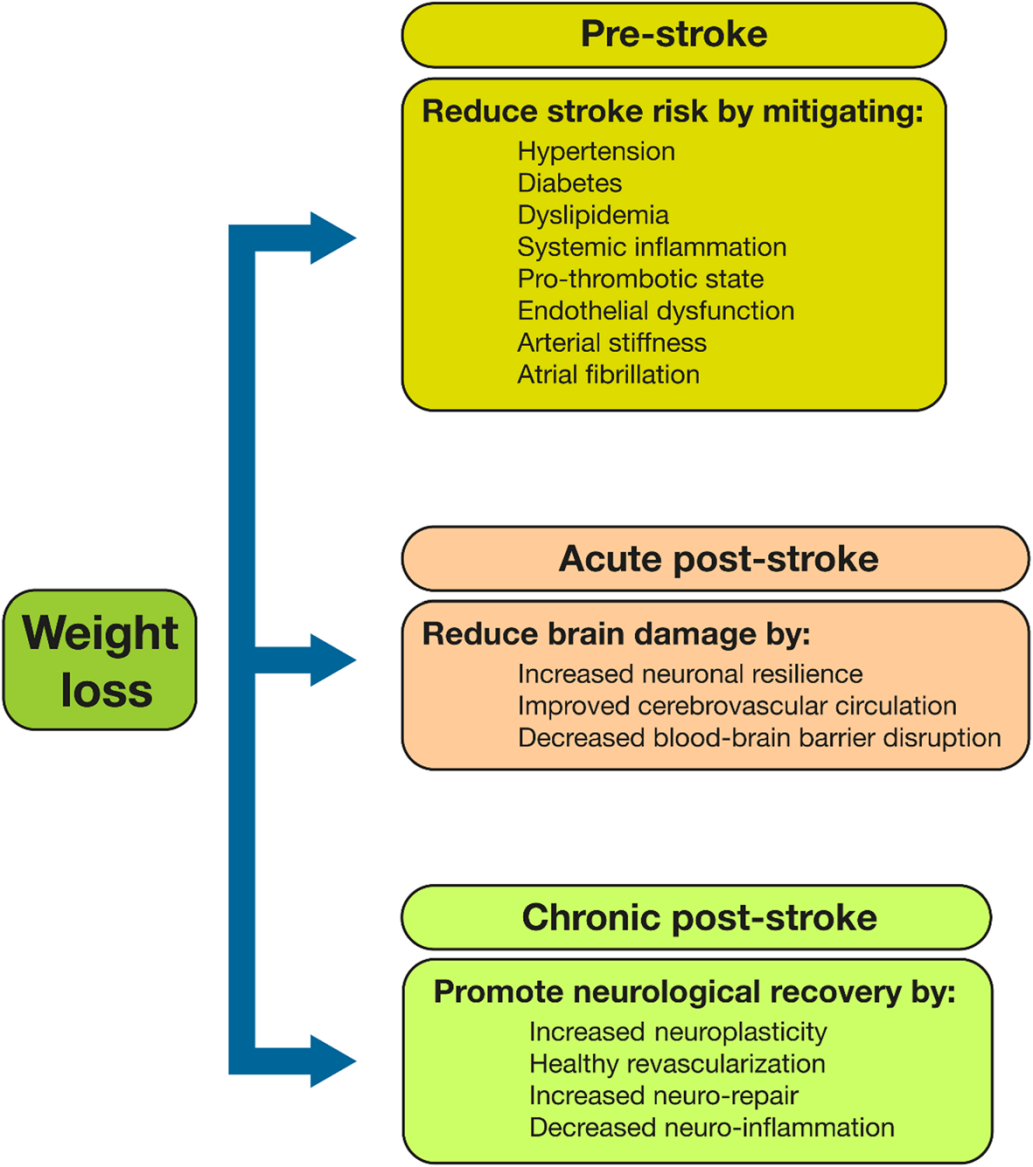
Mechanisms through which weight loss may reduce stroke risk pre-stroke and improve functional outcome after stroke

The clinical studies discussed above indicate that weight loss via lifestyle changes, bariatric surgery, or GLP-1RA reduces CV events in T2D. Despite some contradictory results, a recent systemic review and meta-analysis showed that inducing weight loss can reduce stroke risk [38]. However, reducing stroke risk in T2D is conceptually different from improving functional outcome, thus reducing stroke-related disability. A reduced stroke risk may or may not be driven by the same risk factors and mechanisms that are crucial to improving stroke-induced disability, as schematically summarized in Fig. 1. Specifically, stroke risk reduction primarily addresses systemic risk factors such as hyperglycemia, hypertension, dyslipidemia, and obesity, whereas improving functional outcome after stroke targets mechanisms directly involved in brain injury and subsequent repair, including neuronal survival, neuroplasticity, and neurovascular remodeling [46]. Consequently, interventions that lower stroke risk may not necessarily result in improved functional outcome once stroke occurs. Additionally, improvements in functional outcomes after stroke may be partly mediated by a reduction in the severity of strokes that still occur despite risk-mitigating interventions, such as oral anticoagulation in atrial fibrillation [47]. Whether such a stroke severity-reducing effect may be present also after weight loss is presently unknown.

We recently showed in an animal model of T2D and obesity that diet-induced weight loss before experimental stroke improves neurological outcome [48]. The weight loss needed to be sustained and accompanied by the normalization of hyperglycemia and insulin resistance, since a similar weight loss achieved more rapidly and without normalization of glucose metabolism failed to improve the neurological outcome. In the same T2D/obese mouse model, we also showed that pre-stroke weight loss via GLP-1RA or glucagon-receptor agonists improved neurological outcome after stroke [49, 50]. This effect was entirely mediated by weight loss [50]. While these findings suggest that pre-stroke weight loss improves neurological outcome after stroke in T2D, they were obtained in a single rodent model of stroke and obesity-induced diabetes using only male animals and do not entirely capture the substantial clinical heterogeneity of stroke and diabetes. In addition, translating a strictly pre-stroke weight-loss intervention to patients poses practical challenges. Therefore, additional studies across diverse rodent models and treatment paradigms, in both male and females, are warranted to determine the robustness and generalizability of these results.

Based on the lack of clinical studies addressing whether weight loss improves functional outcomes after stroke in T2D and promising animal studies supporting the potential benefit of weight loss, clinical studies addressing this question are very much needed.

Such studies could be designed to evaluate:

1. The efficacy of weight loss interventions—including lifestyle modification, pharmacotherapy, and bariatric surgery—in improving functional outcomes (e.g., via 90 day modified Rankin Scale) in patients with T2D who suffer a stroke as well as the duration and magnitude of weight loss needed to achieve these improved functional outcomes.
2. The association of weight loss interventions with any reduction in acute severity of subsequent stroke.
3. The role of metabolic normalization, assessing whether improvements in glycemic control, insulin sensitivity, and other metabolic parameters are necessary to mediate beneficial and neurorestorative effects after stroke.
4. Optimal timing and sustainability of interventions—investigating whether pre-stroke weight management is superior to post-stroke interventions, and how the duration and magnitude of weight loss influence functional outcomes.
5. Mechanistic biomarkers—identifying neuroimaging, biochemical, and functional markers that can link weight loss and metabolic improvement to neurorestorative effects and brain repair processes.

The design of such clinical studies could include randomized controlled trials comparing structured weight loss interventions with standard care and should be conducted in specific target populations such as T2D patients with obesity as they are at high risk of ischemic stroke but also those with T2D and obesity who suffered from transient ischemic attack (TIA) who have an increased risk of ischemic stroke. Outcomes should include validated functional scales, quality-of-life measures, and mechanistic biomarkers to elucidate the pathways through which weight loss may improve stroke outcome. Because U- and J-shaped associations between weight loss and adverse cardiovascular outcomes arise from both intentional and unintentional weight loss [51], future studies on weight loss and functional outcome after stroke should account for reverse causality to avoid misattributing poor outcomes to weight loss itself. Filling this knowledge gap could potentially reduce stroke-related disability in T2D and refine the broader understanding of how metabolic interventions influence brain resilience and recovery following cerebrovascular events.

## Conclusions

T2D increases stroke risk and worsens outcomes after stroke. Evidence that weight loss reduces CV risk in T2D suggests that it may also reduce stroke risk. In particular, because intentional weight loss may improve functional outcome after stroke through biological pathways that differ from the mechanisms underlying the obesity paradox (see above), for instance by reducing high blood pressure [52] and chronic low-grade inflammation [53], improving endothelial function [54] and normalizing insulin resistance and metabolic impairments [55]. However, clinical studies investigating whether pre-stroke weight loss can improve outcomes after stroke in T2D are currently lacking and should be conducted. Preferably, this would encompass both legacy studies, evaluating functional outcomes in participants of recently published weight loss trials who have suffered a stroke, and new randomized trials in high-risk stroke populations with T2D. Positive findings from such studies would highlight the prophylactic value of targeting obesity before stroke to minimize stroke *sequelae*, reducing disability. This could have major implications for benefiting a large group of people and reducing the burden of stroke to both individuals and society.

## Data Availability

No datasets were generated or analysed during the current study.

## References

[1] Megherbi S-E, Milan C, Minier D, et al. Association between diabetes and stroke subtype on survival and functional outcome 3 months after stroke: data from the European BIOMED Stroke Project. Stroke. 2003;34:688–94. 12624292 10.1161/01.STR.0000057975.15221.40

[2] Ullberg T, Zia E, Petersson J, et al. Changes in functional outcome over the first year after stroke: an observational study from the Swedish stroke register. Stroke. 2015;46:389–94. 25538204 10.1161/STROKEAHA.114.006538

[3] Echouffo-Tcheugui JB, Xu H, Matsouaka RA, et al. Diabetes and long-term outcomes of ischaemic stroke: findings from get with the guidelines-stroke. Eur Heart J. 2018. 10.1093/eurheartj/ehy036. 29438515 10.1093/eurheartj/ehy036 PMC6031049

[4] Mosenzon O, Cheng AYY, Rabinstein AA, et al. Diabetes and stroke: what are the connections? J Stroke. 2023;25:26–38. 36592968 10.5853/jos.2022.02306 PMC9911852

[5] Bailey RR, Serra MC, McGrath RP. Obesity and diabetes are jointly associated with functional disability in stroke survivors. Disabil Health J. 2020;13:100914. 32139319 10.1016/j.dhjo.2020.100914 PMC7387192

[6] Bailey RR, Conroy M. Diabetes and obesity are associated with disability in community-dwelling stroke survivors: a cross-sectional study of 37,955 Behavioral Risk Factor Surveillance System respondents. Top Stroke Rehabil. 2022;29:156–62. 33775239 10.1080/10749357.2021.1904537

[7] Zabala A, Darsalia V, Holzmann MJ, et al. Risk of first stroke in people with type 2 diabetes and its relation to glycaemic control: a nationwide observational study. Diabetes Obes Metab. 2020;22:182–90. 31576643 10.1111/dom.13885

[8] Kissela BM, Khoury J, Kleindorfer D, et al. Epidemiology of ischemic stroke in patients with diabetes: the greater Cincinnati/Northern Kentucky Stroke Study. Diabetes Care. 2005;28:355–9. 15677792 10.2337/diacare.28.2.355

[9] Mavridis A, Viktorisson A, Eliasson B, et al. Risk of ischemic and hemorrhagic stroke in individuals with type 1 and type 2 diabetes: a nationwide cohort study in Sweden. Neurology. 2025;104:e213480. 40080734 10.1212/WNL.0000000000213480 PMC11907640

[10] Cho NH, Shaw JE, Karuranga S, et al. IDF Diabetes Atlas: Global estimates of diabetes prevalence for 2017 and projections for 2045. Diabetes Res Clin Pract. 2018;138:271–81. 29496507 10.1016/j.diabres.2018.02.023

[11] Powell-Wiley TM, Poirier P, Burke LE, et al. Obesity and cardiovascular disease a scientific statement from the American Heart Association. Circulation. 2021;143:E984–1010. 33882682 10.1161/CIR.0000000000000973 PMC8493650

[12] Strazzullo P, D’Elia L, Cairella G, et al. Excess body weight and incidence of stroke: meta-analysis of prospective studies with 2 million participants. Stroke. 2010;41:e418-426. 20299666 10.1161/STROKEAHA.109.576967

[13] Li W, Katzmarzyk PT, Horswell R, et al. Body mass index and stroke risk among patients with type 2 diabetes mellitus. Stroke. 2015;46:164–9. 25468880 10.1161/STROKEAHA.114.006718 PMC4276457

[14] Qin J, Zhang T, Chen Y, et al. The effect of body mass index on stroke prognosis: A systematic review and meta-analysis of 32 cohort studies with 330,353 patients. Int J Stroke. 2024;19:1093–101. 38699977 10.1177/17474930241255031

[15] Rodríguez-Castro E, Rodríguez-Yáñez M, Arias-Rivas S, et al. Obesity paradox in ischemic stroke: clinical and molecular insights. Transl Stroke Res. 2019;10:639–49. 30980283 10.1007/s12975-019-00695-x

[16] Lee SH, Jung JM, Park MH. Obesity paradox and stroke outcomes according to stroke subtype: a propensity score-matched analysis. Int J Obes (Lond). 2023;47:669–76. 37137958 10.1038/s41366-023-01318-0

[17] Oesch L, Tatlisumak T, Arnold M, et al. Obesity paradox in stroke - Myth or reality? A systematic review PLoS One. 2017;12:e0171334. 28291782 10.1371/journal.pone.0171334 PMC5349441

[18] Prillaman M. Beyond BMI: how to redefine obesity. Nature. 2023;622:232–3. 10.1038/d41586-023-03257-2 37903933

[19] Rubino F, Cummings DE, Eckel RH, et al. Definition and diagnostic criteria of clinical obesity. Lancet Diabetes Endocrinol. 2025;13:221–62. 39824205 10.1016/S2213-8587(24)00316-4 PMC11870235

[20] Lu J, Gong S, Zhu J, et al. Relationships between obesity and functional outcome after ischemic stroke: a Mendelian randomization study. Neurol Sci. 2024;45:3869–77. 38466476 10.1007/s10072-024-07415-w

[21] Wang M, Daghlas I, Aldridge CM, et al. Adiposity and domain-specific stroke recovery: a Mendelian randomization study. Eur Stroke J. 2025;10:940–5. 39953957 10.1177/23969873251319916 PMC11830152

[22] Liu Z, Sanossian N, Starkman S, et al. Adiposity and Outcome After Ischemic Stroke: Obesity Paradox for Mortality and Obesity Parabola for Favorable Functional Outcomes. Stroke. 2021;52:144–51. 33272129 10.1161/STROKEAHA.119.027900

[23] GBD 2021 Stroke Risk Factor Collaborators. Global, regional, and national burden of stroke and its risk factors, 1990-2021: a systematic analysis for the Global Burden of Disease Study 2021. Lancet Neurol. 2024;23:973–1003. 39304265 10.1016/S1474-4422(24)00369-7 PMC12254192

[24] Knowler WC, Barrett-Connor E, Fowler SE, et al. Reduction in the incidence of type 2 diabetes with lifestyle intervention or metformin. N Engl J Med. 2002;346:393–403. 11832527 10.1056/NEJMoa012512 PMC1370926

[25] Lean ME, Leslie WS, Barnes AC, et al. Primary care-led weight management for remission of type 2 diabetes (DiRECT): an open-label, cluster-randomised trial. Lancet. 2018;391:541–51. 29221645 10.1016/S0140-6736(17)33102-1

[26] Svanevik M, Lorentzen J, Borgeraas H, et al. Patient-reported outcomes, weight loss, and remission of type 2 diabetes 3 years after gastric bypass and sleeve gastrectomy (Oseberg); a single-centre, randomised controlled trial. Lancet Diabetes Endocrinol. 2023;11:555–66. 37414071 10.1016/S2213-8587(23)00127-4

[27] Arterburn DE, Telem DA, Kushner RF, et al. Benefits and Risks of Bariatric Surgery in Adults: A Review. JAMA. 2020;324:879–87. 32870301 10.1001/jama.2020.12567

[28] Carlsson LMS, Peltonen M, Ahlin S, et al. Bariatric Surgery and Prevention of Type 2 Diabetes in Swedish Obese Subjects. N Eng J Med. 2012;367:695–704. 10.1056/NEJMoa1112082 22913680

[29] Vieira FT, Prado CM, Thorlakson J, et al. Sarcopenic Obesity in Metabolic and Bariatric Surgery: A Scoping Review. Obes Rev. 2025;26:e13973. 40556340 10.1111/obr.13973 PMC12620107

[30] Sanchis-Gomar F, Neeland IJ, Lavie CJ. Balancing weight and muscle loss in GLP1 receptor agonist therapy. Nat Rev Endocrinol. 2025;21:584–5. 40721501 10.1038/s41574-025-01160-6

[31] Villareal DT, Aguirre L, Gurney AB, et al. Aerobic or Resistance Exercise, or Both, in Dieting Obese Older Adults. N Eng J Med. 2017;376:1943–55. 10.1056/NEJMoa1616338 PMC5552187 28514618

[32] Bellicha A, Ciangura C, Poitou C, et al. Effectiveness of exercise training after bariatric surgery—a systematic literature review and meta-analysis. Obes Rev. 2018;19:1544–56. 30156007 10.1111/obr.12740

[33] Association of the magnitude of weight loss and changes in physical fitness with long-term cardiovascular disease outcomes in overweight or obese people with type 2 diabetes: a post-hoc analysis of the Look AHEAD randomised clinical trial. Lancet Diabetes Endocrinol 2016;4:913–921. 10.1016/S2213-8587(16)30162-0 PMC5094846 27595918

[34] Gregg EW, Chen H, Bancks MP, et al. Impact of remission from type 2 diabetes on long-term health outcomes: findings from the Look AHEAD study. Diabetologia. 2024;67:459–69. 38233592 10.1007/s00125-023-06048-6 PMC10844408

[35] Sattar N, Taheri S, Astling DP, et al. Prediction of cardiometabolic health through changes in plasma proteins with intentional weight loss in the direct and diadem-i randomized clinical trials of type 2 diabetes remission. Diabetes Care. 2023;46:1949–57. 37756566 10.2337/dc23-0602 PMC10628468

[36] Lean ME, Leslie WS, Barnes AC, et al. 5-year follow-up of the randomised Diabetes Remission Clinical Trial (DiRECT) of continued support for weight loss maintenance in the UK: an extension study. Lancet Diabetes Endocrinol. 2024;12:233–46. 38423026 10.1016/S2213-8587(23)00385-6

[37] Khater A, Al-Badri M, Salah T, et al. Long-Term Effects of Intensive Lifestyle Intervention on Cardiometabolic Outcomes in Patients With Diabetes in Real-World Clinical Practice: A 15-Year Longitudinal Study. J Diabetes. 2025;17:e70153. 41192942 10.1111/1753-0407.70153 PMC12588724

[38] Wang X, Huang Y, Chen Y, et al. The relationship between body mass index and stroke: a systemic review and meta-analysis. J Neurol. 2022;269:6279–89. 35971008 10.1007/s00415-022-11318-1

[39] Drucker DJ. Mechanisms of action and therapeutic application of Glucagon-like Peptide-1. Cell Metab. 2018;27:740–56. 29617641 10.1016/j.cmet.2018.03.001

[40] Marso SP, Bain SC, Consoli A, et al. Semaglutide and Cardiovascular Outcomes in Patients with Type 2 Diabetes. N Eng J Med. 2016;375:1834–44. 10.1056/NEJMoa1607141 27633186

[41] Lincoff AM, Brown-Frandsen K, Colhoun HM, et al. Semaglutide and Cardiovascular Outcomes in Obesity without Diabetes. N Eng J Med. 2023;389:2221–32. 10.1056/NEJMoa2307563 37952131

[42] Hernandez AF, Green JB, Janmohamed S, et al. Albiglutide and cardiovascular outcomes in patients with type 2 diabetes and cardiovascular disease (Harmony Outcomes): a double-blind, randomised placebo-controlled trial. Lancet. 2018;392:1519–29. 30291013 10.1016/S0140-6736(18)32261-X

[43] Deanfield J, Lincoff AM, Kahn SE, et al. Semaglutide and cardiovascular outcomes by baseline and changes in adiposity measurements: a prespecified analysis of the SELECT trial. Lancet. 2025;406:2257–68. 41138739 10.1016/S0140-6736(25)01375-3

[44] Michaelsen MK, Drasbek KR, Valentin JB, et al. GLP-1 receptor agonists as treatment of nondiabetic ischemic stroke—a systematic review and meta-analysis. Stroke. 2026;57:415–37. 41263069 10.1161/STROKEAHA.125.053075

[45] Darsalia V, Klein T, Nyström T, et al. Glucagon-like receptor 1 agonists and DPP-4 inhibitors: anti-diabetic drugs with anti-stroke potential. Neuropharmacology. 2018;136:280–6. 28823610 10.1016/j.neuropharm.2017.08.022

[46] Krinock MJ, Singhal NS. Diabetes, stroke, and neuroresilience: looking beyond hyperglycemia. Ann N Y Acad Sci. 2021;1495:78–98. 33638222 10.1111/nyas.14583

[47] Meinel TR, Branca M, De Marchis GM, et al. Prior Anticoagulation in Patients with Ischemic Stroke and Atrial Fibrillation. Ann Neurol. 2021;89:42–53. 32996627 10.1002/ana.25917 PMC7756294

[48] Karampatsi D, Zabala A, Wilhelmsson U, et al. Diet-induced weight loss in obese/diabetic mice normalizes glucose metabolism and promotes functional recovery after stroke. Cardiovasc Diabetol. 2021;20:1–16. 34937562 10.1186/s12933-021-01426-z PMC8697500

[49] Vercalsteren E, Karampatsi D, Neicu M, et al. Normalization of Insulin Resistance, Rather Than Hyperglycemia, Before Stroke Improves Functional Outcomes in a Mouse Model of Type 2 Diabetes. Diabetes 2026;db250532. 10.2337/db25-0532. Online ahead of print. PMID: 41805868. 10.2337/db25-0532 PMC13097208 41805868

[50] Vercalsteren E, Karampatsi D, Neicu M, et al. Pre-stroke weight loss by glucagon-like peptide 1 receptor and neuropeptide Y receptor Y2 activation improves post-stroke functional recovery in male diabetic mouse models. Diabetologia. 2026;69:230–43. 41094027 10.1007/s00125-025-06567-4 PMC12686085

[51] Park CS, Choi YJ, Rhee TM, et al. U-Shaped Associations Between Body Weight Changes and Major Cardiovascular Events in Type 2 Diabetes Mellitus: A Longitudinal Follow-up Study of a Nationwide Cohort of Over 1.5 Million. Diabetes Care. 2022:45:1239–1246. 10.2337/dc21-2299 35263435

[52] Schiavon CA, Cavalcanti AB, Oliveira JD, et al. Randomized trial of effect of bariatric surgery on blood pressure after 5 years. J Am Coll Cardiol. 2024;83:637–48. 38325988 10.1016/j.jacc.2023.11.032

[53] Forsythe LK, Wallace JMW, Livingstone MBE. Obesity and inflammation: the effects of weight loss. Nutr Res Rev. 2008;21:117–33. 19087366 10.1017/S0954422408138732

[54] Li X, Wang S, Sun L, et al. Effects of bariatric surgery on endothelial function and structure in individuals with obesity: a prospective study. Obes Surg. 2025;35:3082–92. 40632456 10.1007/s11695-025-08004-2

[55] Patikorn C, Roubal K, Veettil SK, et al. Intermittent fasting and obesity-related health outcomes: an umbrella review of meta-analyses of randomized clinical trials. JAMA Netw Open. 2021;4:e2139558. 34919135 10.1001/jamanetworkopen.2021.39558 PMC8683964

